# Supplementary material for: The interactome of intact mitochondria by cross-linking mass spectrometry provides evidence for coexisting respiratory supercomplexes
Source: Mol Cell Proteomics. 2017 Dec 8;17(2):216–32. doi: 10.1074/mcp.RA117.000470 (PMC5795388; doi:10.1074/mcp.RA117.000470)
Supplement: Supplemental Data [file supp_RA117.000470_133922_0_supp_23978_fzffwf.zip › spectra_annotation/mito_spectra_annotation/39-2-6-1-14-1.pdf]

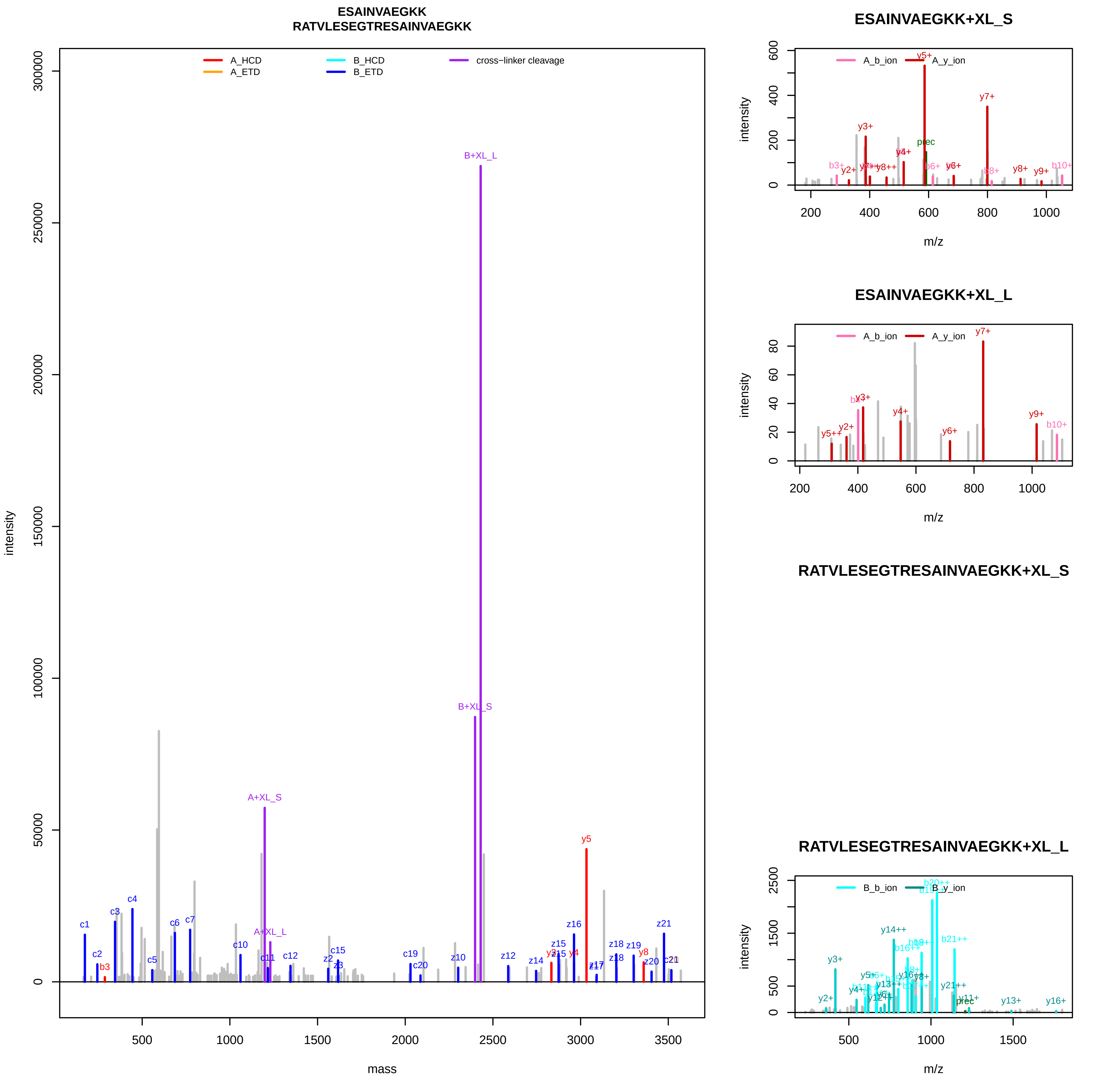

ESAINVAEGKK
RATVLESEGTRESAINVAEGKK
A_HCD
A_ETD
B_HCD
B_ETD
cross−linker cleavage
B+XL_L
B+XL_S
A+XL_S
A+XL_L
c1
c2
b3
c3
c4
c5
c6
c7
c10
c11
c12
c15
c19
c20
z10
z12
z14
z15
z16
z17
z18
z19
z20
z21
c21
y5
y8
intensity
mass
ESAINVAEGKK+XL_S
ESAINVAEGKK+XL_L
RATVLESEGTRESAINVAEGKK+XL_S
RATVLESEGTRESAINVAEGKK+XL_L
A_b_ion
A_y_ion
B_b_ion
B_y_ion
prec
y3+
y4+
y5+
y6+
y7+
y8+
y9+
b3+
b10+
y14++
y21++
y2+
y13+
y16+
m/z
